# Differences in healthcare service utilization in patients with polypharmacy according to their risk level by adjusted morbidity groups: a population-based cross-sectional study

**DOI:** 10.1186/s40545-023-00665-7

**Published:** 2023-11-28

**Authors:** Jaime Barrio-Cortes, Beatriz Benito-Sánchez, Ana Isabel Villimar-Rodriguez, Miguel Rujas, Peña Arroyo-Gallego, Jim Carlson, Beatriz Merino-Barbancho, Ana Roca-Umbert, Andrés Castillo-Sanz, Francisco Lupiáñez-Villanueva, Giuseppe Fico, Tomás Gómez-Gascón

**Affiliations:** 1 Foundation for Biosanitary Research and Innovation in Primary Care (FIIBAP), Ave. Reina Victoria, 21, 6Th Floor, 28003 Madrid, Spain; 2 Faculty of Health, Camilo José Cela University, Madrid, Spain; 3 Gregorio Marañón Health Research Institute, Madrid, Spain; 4 Research Network On Chronicity, Primary Care and Prevention and Health Promotion, Carlos III Health Institute, Madrid, Spain; 5 Primary Care Pharmacy, Care Management of the Central Territorial Area, Madrid Health Service, Madrid, Spain; 6 Technical University of Madrid (UPM), Madrid, Spain; 7 Predictby, Barcelona, Spain; 8 Foundation for Research of Hospital Infantil Niño Jesús, Madrid, Spain; 9 Research Institute Hospital, 12 de Octubre (imas12), Primary Care Management, Madrid, Spain; 10 Faculty of Medicine, Complutense University of Madrid, Madrid, Spain

**Keywords:** Polypharmacy, Chronic disease, Primary care, Hospital care, Healthcare utilization, Management, Morbidity grouper, Stratification

## Abstract

**Background:**

Patients with polypharmacy suffer from complex medical conditions involving a large healthcare burden. This study aimed to describe the characteristics and utilization of primary care (PC) and hospital care (HC) and factors associated in chronic patients with polypharmacy, stratifying by adjusted morbidity groups (AMG) risk level, sex and age, and comparing with non-polypharmacy.

**Methods:**

Cross-sectional study conducted in a Spanish basic healthcare area. Studied patients were those over 18 years with chronic diseases identified by the AMG tool from Madrid electronic clinical record, which was the data source. Sociodemographic, sociofunctional, clinical and healthcare utilization variables were described and compared by risk level, sex, age and having or not polypharmacy. Factors associated with healthcare utilization in polypharmacy patients were determined by a negative binomial regression model.

**Results:**

In the area studied, 61.3% patients had chronic diseases, of which 16.9% had polypharmacy vs. 83.1% without polypharmacy. Patients with polypharmacy (vs. non-polypharmacy) mean age was 82.7 (vs. 52.7), 68.9% (vs. 60.7%) were women, and 22.0% (vs. 1.2%) high risk. Their average number of chronic diseases was 4.8 (vs. 2.2), and 95.6% (vs. 56.9%) had multimorbidity. Their mean number of annual healthcare contacts was 30.3 (vs. 10.5), 25.9 (vs. 8.8) with PC and 4.4 (vs. 1.7) with HC. Factors associated with a greater PC utilization in patients with polypharmacy were elevated complexity, high risk level and dysrhythmia. Variables associated with a higher HC utilization were also increased complexity and high risk, in addition to male sex, being in palliative care, having a primary caregiver, suffering from neoplasia (specifically lymphoma or leukaemia) and arthritis, whereas older age and immobilization were negatively associated.

**Conclusions:**

Polypharmacy population compared to non-polypharmacy was characterized by a more advanced age, predominance of women, high-risk, complexity, numerous comorbidities, dependency and remarkable healthcare utilization. These findings could help healthcare policy makers to optimize the distribution of resources and professionals within PC and HC systems, aiming for the improvement of polypharmacy management and rational use of medicines while reducing costs attributed to healthcare utilization by these patients.

**Supplementary Information:**

The online version contains supplementary material available at 10.1186/s40545-023-00665-7.

## Background

Polypharmacy is defined as the concurrent use of multiple medications according to the World Health Organization (WHO) [1]. Despite the increasing prevalence of polypharmacy, there is no universal definition of polypharmacy. All the studies conducted on this topic are subject to this inevitable bias, making it challenging to assess the extent of the problem and the impact on relevant health outcomes [2]. A narrative review of the recent publications on this subject found that the majority of definitions were mere numerical, varying the threshold from two to 11 or more medications, some including associated terms to define the level of polypharmacy, others classifying the duration of drug treatment or indicating the health care setting, and there are also purely descriptive definitions [3].

The incidence of polypharmacy is continuously rising worldwide on account of the increase in life expectancy, the growing prevalence of chronic diseases and multimorbidity and the expanding variety of available treatments [2]. In Spain, the prevalence of patients taking at least five drugs more than tripled in 10 years [4]. The prevalence of polypharmacy greatly varies from less than 10% to more than 90%, depending on the age group, the definition applied and the healthcare and geographical setting [3]. A recent systematic review and meta-analysis reported a pooled estimated prevalence of polypharmacy in all medication classes of 37% [5].

Numerous factors underlie polypharmacy and are markedly different in non-polypharmacy patients. These factors include the ones linked to the patient (age, sex, education, socioeconomic status, location of residence, ethnicity and behaviour), disease-related factors (multimorbidity, frailty and certain conditions, such as cardiovascular, metabolic and respiratory illnesses), and factors linked to the healthcare system or to the physician [6]. Exposure to a higher number of medicines contribute to the development of the known inappropriate polypharmacy that emerges when more drugs are prescribed than necessary, as well as when a necessary drug is prescribed incorrectly and when a clinically indicated drug is not prescribed [5]. This common critical situation among patients with polypharmacy represents a major concern that leads to negative health outcomes, such as adverse drug effects, harmful drug interactions, medication nonadherence, functional and cognitive decline, frailty, higher use of healthcare services, increased risk of hospitalization, longer hospital stays, higher mortality, and errors in transitions of care [3, 5]. Therefore, a suitable intervention model is needed, involving the redesign of care processes and services, to improve patient-oriented outcomes and adaptation of the therapeutic regimen to the reality of patients, with special focus on polypharmacy [1].

The WHO holds that polypharmacy management involves multifaceted decision-making and necessitates the combined knowledge of health care professionals, requiring coordination between primary care (PC) and hospital care (HC) to ensure a continuum of care focused on the patient. Multiple programmes have been implemented to address polypharmacy, some of them focused on older patients with multimorbidity [1]. On the other hand, different multimorbidity tools have been developed around the world to assist in the management of patients with multimorbidity [7]. In Spain, the Adjusted Morbidity Groups (AMG) had been devised and adapted to the Spanish healthcare environment, allowing the general population to be stratified into risk groups based on comorbidity and complexity measurements obtained from their clinical history [8]. The AMG aggregator was created to inform clinicians and healthcare policy makers of the use of healthcare resources and to guide them in their decision-making, aiming to achieve an efficient and personalized model of care focused on case and disease management and resource planning, depending on the specific socioclinical needs of each individual patient [7, 8].

In comparison with the patients without polypharmacy, those with polypharmacy are more susceptible to poor adherence to treatment [1], which is the reason why they are being studied within the framework of the IMI-H2020 European project—“BEAMER” (“BEhavioral and Adherence Model for improving quality, health outcomes and cost-effectiveness of healthcaRe”). BEAMER was created with the aim of addressing the existing problems derived from non-adherence to medication. For achieving its purpose, a model that segments the population based on actionable factors and predicts adherence behaviour is going to be developed, to help build new digital health solutions and promote better outcomes for patients, healthcare systems and the pharmaceutical industry. BEAMER model will be created starting from the data of polypharmacy patients of different European populations, and at this point, the present study is providing the initial data analysed from a region of Madrid, in Spain [9].

Besides, the AMG system has been implemented in recent years by the Ministry of Health into the PC digital health records of several Autonomous Communities in Spain and to date they have never been specifically studied in chronic patients with polypharmacy. Therefore, we aimed to describe the characteristics and utilization of PC and HC services by AMG risk level, sex and age, in chronic patients with polypharmacy and the factors associated with their utilization, as well as the differences without polypharmacy, to better understand these populations and their healthcare and pharmaceutical needs.

## Methods

### Study design and setting

This cross-sectional study was conducted in a basic health area in Chamartín, which had a reference hospital in the city of Madrid. On June 30, 2015, this basic health area had a total of 18,107 patients, 15,416 of whom were older than 18. The number of inhabitants in Chamartín was 143,424, characterized by a mean age of 45 years (23% older than 65), 55% being women and 8.9% foreigners.

### Data collection and participant selection

The PC digital clinical record system of the Community of Madrid was the data source used to obtain all the medical information of the patients in the study. Patients included in the study were the entire population of patients registered in the area studied which had at least one of the chronic diseases described in Additional file 1: Table S1*,* who were identified using the AMG tool of PC digital clinical record [8]. Patients younger than 18 years were excluded.

### Variables analysed

The AMG tool calculates a numerical complexity index based on the risk of hospital admission, mortality, visits to PC and pharmacy expenditures. This complexity index allows the stratification of the overall population into low, medium and high risk levels for patients with chronic diseases and one last group for subjects without a chronic condition, corresponding to the cutoff points at the 50th, 85th and 95th percentiles of the population [8].

The variables analysed were I) sociodemographic characteristics: age, sex, country of birth; II) sociofunctional characteristics: immobilized at home, institutionalized in a nursing home, need for a primary caregiver, home support, palliative care; III) clinical data: AMG risk level (high, medium or low risk), complexity index, number and type of chronic diseases, multimorbidity (coexistence of two or more chronic conditions [1]), polypharmacy (prescription of six or more medications, according to the definition of the Community of Madrid [10] and as registered in Madrid electronic clinical record); IV) PC service utilization (measured by the total number of contacts registered with the PC system): total number of contacts per year, type of interaction (medical, administrative, laboratory), way to communicate (on site, by phone, at home), professional consulted (family physician, nurse, social worker, midwife, physiotherapist, dentist); and V) HC service utilization (measured by the total number of contacts registered with the HC system): total number of contacts per year, contact site (outpatient clinic, emergency room visit, admissions, day hospital visit). Sociodemographic, sociofunctional and clinical variables were obtained from the PC electronic clinical record on 30 June 2015, and healthcare resource utilization over 1 year, from 30 June 2015 to 30 June 2016.

### Data analysis

Data analysis was performed on the entire population with chronic diseases, also on the resulting subgroups after segmenting by having or not polypharmacy, and additionally on the subpopulations identified by dividing the polypharmacy population by sex, age and risk level.

The entire population with chronic diseases and the subpopulations were characterized by calculating the descriptive statistics of all the variables under study. Categorical variables are defined as counts and percentages, while quantitative data are presented as mean with standard deviation or median with interquartile range. Normality was tested with the Shapiro‒Wilk test. Bivariate analyses were performed by applying the chi‐squared test or Fisher's exact test when appropriate to compare categorical variables, whereas parametric and nonparametric tests were used for polytomous and quantitative variables, according to their distribution. Multiple-comparison results were adjusted with the Bonferroni correction. Considering that healthcare utilization is measured as nonnegative data that exhibit substantial positive skewness and a mass at zero for patients who do not register any encounter with PC or HC, we built a negative binomial regression model to identify the factors associated with the use of PC and HC [11]. Results from bivariate and multivariate comparisons were considered statistically significant when *p* < 0.05. The statistical analysis was performed with IBM SPSS Statistics version 25 (IBM Corp., Armonk, NY).

## Results

Among the 15,416 patients over 18 years of the health area studied, 9443 (61.3%) patients had chronic diseases, of which 1598 (16.9%) had polypharmacy and 7845 (83.1%) were patients without polypharmacy. Of the population with polypharmacy, 68.9% were women and the mean age was 82.7 years, being, respectively, 60.7% and 52.7 years for the individuals without polypharmacy. Considering the sociofunctional variables, patients with polypharmacy showed a notably higher level of limitations than patients without polypharmacy. Following the AMG system to stratify the clinical data and contrasting individuals with vs. without polypharmacy, 29.6% vs. 86.1% were classified as low risk, 48.4% vs. 12.7% as medium risk and 22% vs. 1.2% as high risk, accounting for a mean complexity index of 15.1 vs. 5.2. The mean number of chronic diseases per person was also larger for subjects with polypharmacy (4.8) than the patients without polypharmacy (2.2), and 95.6% vs. 56.9% of each group had multimorbidity. These global characteristics are described in Table 1, showing the results for the overall patients with chronic diseases as well as divided by polypharmacy vs. non-polypharmacy, whereas the polypharmacy results stratified by AMG risk level are shown in Additional file 1: Table S2 and by sex and age group in Additional file 1: Table S3*.*

**Table 1:** Sociodemographic, functional and clinical characteristics of patients with chronic diseases and with or without polypharmacy

*n* (%)	Total patients with chronic diseases 9443 (100%)	95% CI	Non-polypharmacy patients 7845 (83.1%)	95% CI	Polypharmacy patients 1598 (16.9%)	95% CI	*p*
Sociodemographic variables							
Female	5862 (62.1%)	61.1–63.1	4,761 (60.7%)	59.6–61.8	1,101 (68.9%)	66.6–71.2	< 0.01
Age*	57.6 [18.7]	57.4–58.2	52.7 [16.1]	52.4–53.1	82.7 [7.1]	82.4–83.1	< 0.01
≤ 75 years	7473 (79.1%)	78.3–80.0	7,318 (93.3%)	92.7–93.8	155 (9.7%)	8.3–11.2	< 0.01
> 75 years	1970 (20.9%)	20.0–21.68	527 (6.7%)	6.2–7.3	1,443 (90.3%)	88.9–91.8	
Origin Spain	7762 (82.2%)	81.4–83.0	6,381 (81.3%)	80.5–82.2	1,381 (86.4%)	84.7–88.1	< 0.01
Rest of Europe	346 (3.7%)	3.3–4.0	300 (3.8%)	3.4–4.2	46 (2.9%)	2.1–3.7	
Rest of the world	1335 (14.1%)	13.4–14.8	1,164 (14.8%)	14.1–15.6	171 (10.7%)	9.2–12.2	
Functional variables							
Immobilized	300 (3.2%)	2.8–3.5	43 (0.5%)	0.4–0.7	257 (16.1%)	14.3–17.9	< 0.01
Institutionalized	161 (1.7%)	1.4–2.0	45 (0.6%)	0.4–0.7	116 (7.3%)	6.0–8.5	< 0.01
Primary caregiver	228 (2.4%)	2.1–2.7	28 (0.4%)	0.1–0.5	200 (12.5%)	10.9–14.1	< 0.01
Home support	80 (0.8%)	0.7–1.0	9 (0.1%)	0.04–0.2	71 (4.4%)	3.4–5.5	< 0.01
Palliative care	44 (0.5%)	0.3–0.6	14 (0.2%)	0.09–0.3	30 (1.9%)	1.2–2.5	< 0.01
Clinical variables							
AMG Risk Level High	443 (4.7%)	4.3–5.1	92 (1.2%)	0.9–1.4	351 (22.0%)	19.9–24.0	< 0.01
Medium	1770 (18.7%)	18.0–19.5	996 (12.7%)	12.0–13.4	774 (48.4%)	46.0–50.9	
Low	7230 (76.6%)	75.7–77.4	6,757 (86.1%)	85.4–86.9	473 (29.6%)	27.3–31.8	
Complexity index*	6.9 [7.1]	6.7–7.0	5.2 [4.5]	5.1–5.3	15.1 [10.9]	14.6–15.6	< 0.01
Chronic diseases*	2.6 [1.8]	2.56–2.63	2.2 [1.4]	2.1–2.2	4.8 [2.2]	4.64–4.86	< 0.01
Multimorbidity	5991 (63.4%)	62.5–64.4	4,463 (56.9%)	55.8–58.0	1,528 (95.6%)	94.6–96.6	< 0.01

^*^Mean [standard deviation]

*AMG* Adjusted Morbidity Groups; *CI* Confidence Interval

Patients with polypharmacy had significantly more comorbidities than patients without polypharmacy. The most prevalent comorbidities within the population with polypharmacy were hypertension (80.4%), dyslipidaemia (64.0%), diabetes (29.0%), osteoporosis (25.4%), dysrhythmias (24.8%), arthrosis (24.2%), obesity (24.0%), and thyroid disorder (22.0%). A total of 193 patients with polypharmacy (12.1%) had at least one active neoplasia, most frequently affecting the prostate (7.0% of men), breast (1.8%), skin (1.5%), colorectum (1.4%) and bladder (1.3%). The main comorbidities in patients with chronic diseases and divided by having polypharmacy or not are described in Table 2, and for polypharmacy patients by AMG risk level and by sex and age group in Additional file 1: Tables S4 and S5*,* respectively*.*

**Table 2:** Comorbidities in patients with chronic diseases and with or without polypharmacy

*n* (%)	Total patients with chronic diseases 9443 (100%)	95% CI	Non-polypharmacy patients 7845(83.1%)	95% CI	Polypharmacy patients 1598 (16.9%)	95% CI	*p*
Haematic comorbidities							
Anaemia	863 (9.1%)	8.6–9.7	671 (8.6%)	7.9–9.2	192 (12.0%)	10.4–13.6	< 0.01
HIV	54 (0.6%)	0.4–0.7	52 (0.7%)	0.5–0.8	2 (0.1%)	0.0–0.3	< 0.01
Digestive comorbidities							
Cirrhosis	476 (5.0%)	4.6–5.5	365 (4.7%)	4.2–5.1	111 (6.9%)	5.7–8.2	< 0.01
Inflammatory bowel disease	74 (0.8%)	0.6–0.9	62 (0.8%)	0.6–1.0	12 (0.8%)	0.3–1.2	0.87
Gastrointestinal ulcer	175 (1.9%)	1.6–2.1	128 (1.6%)	1.4–1.9	47 (2.9%)	2.1–3.8	< 0.01
Chronic pancreatitis	8 (0.1%)	0.0–0.1	5 (0.1%)	0.0–0.1	3 (0.2%)	0.0–0.4	0.12
Ocular comorbidities							
Glaucoma	385 (4.2%)	3.8–4.6	229 (2.9%)	2.5–3.3	166 (10.4%)	8.9–11.9	< 0.01
Cardiovascular comorbidities							
Hypertension	3415 (36.2%)	35.2–37.1	2130 (27.2%)	26.2–28.1	1285 (80.4%)	78.5–82.4	< 0.01
Diabetes Mellitus	1062 (11.2%)	10.6–11.9	599 (7.6%)	7.0–8.2	463 (29.0%)	26.7–31.2	< 0.01
Dyslipidaemia	3757 (39.8%)	38.8–40.8	2734 (34.9%)	33.8–35.9	1023 (64.0%)	61.7–6.4	< 0.01
Dysrhythmias	394 (7.3%)	6.8–7.9	298 (3.8%)	3.4–4.2	396 (24.8%)	22.7–26.9	< 0.01
Heart chronic failure	240 (2.5%)	2.2–2.9	46 (0.6%)	0.4–0.8	194 (12.1%)	10.5–13.7	< 0.01
Ischaemic heart disease	370 (3.9%)	3.5–4.3	153 (2.0%)	1.6–2.3	217 (13.6%)	11.9–15.3	< 0.01
Valvular heart disease	193 (2.0%)	1.8–2.3	93 (1.2%)	0.9–1.4	100 (6.3%)	5.1–7.4	< 0.01
Musculoskeletal comorbidities							
Arthrosis	1055 (11.2%)	10.5–11.8	669 (8.5%)	7.9–9.1	386 (24.2%)	22.1–26.3	< 0.01
Osteoporosis	1,112 (11.8%)	11.1–12.4	706 (9.0%)	8.4–9.6	406 (25.4%)	23.3–27.5	< 0.01
Arthritis	221 (2.3%)	2.0–2.6	160 (2.0%)	1.7–2.4	61 (3.8%)	2.9–4.8	< 0.01
Lupus	5 (0.1%)	0.0–0.01	2 (0.0%)	0.0–0.1	3 (0.2%)	0.0–0.4	0.01
Vasculitis	26 (0.3%)	0.2–0.4	14 (0.2%)	0.1–0.3	12 (0.8%)	0.3–1.2	< 0.01
Neurological comorbidities							
Dementia	213 (2.3%)	2.0–2.6	64 (0.8%)	0.6–1.0	149 (9.3%)	7.9–10.8	< 0.01
Stroke	263 (2.8%)	2.5–3.1	108 (1.4%)	1.1–1.6	155 (9.7%)	8.2–11.2	< 0.01
Parkinson	85 (0.9%)	0.7–1.1	33 (0.4%)	0.3–0.6	52 (3.3%)	2.4–4.1	< 0.01
Epilepsy	167 (1.8%)	1.5–2.0	119 (1.5%)	1.2–1.8	48 (3.0%)	2.2–3.8	< 0.01
Multiple sclerosis	32 (0.3%)	0.2–0.5	31 (0.4%)	0.3–0.5	1 (0.1%)	0.0–0.2	0.04
Psychiatric comorbidities							
Alcohol abuse	406 (4.3%)	3.9–4.7	352 (4.5%)	4.0–4.9	54 (3.4%)	2.5–4.3	0.05
Substance abuse	129 (1.4%)	1.1–1.6	123 (1.6%)	1.3–1.8	6 (0.4%)	0.1–0.7	< 0.01
Anxiety	2,322 (24.6%)	23.7–25.5	2031 (25.9%)	24.9–26.9	291 (18.2%)	16.3–20.1	< 0.01
Depression	1243 (13.2%)	12.5–13.8	932 (11.9%)	11.2–12.6	311 (19.5%)	17.5–21.4	< 0.01
Bipolar disorder	66 (0.7%)	0.5–0.9	56 (0.7%)	0.5–0.9	10 (0.6%)	0.2–1.0	0.07
Psychotic disorder	73 (0.8%)	0.6–0.9	58 (0.7%)	0.5–0.9	15 (0.9%)	0.5–1.4	0.41
Respiratory comorbidities							
COPD	387 (4.1%)	3.7–4.5	198 (2.5%)	2.2–2.9	189 (11.8%)	10.2–13.4	< 0.01
Asthma	880 (9.3%)	8.7–9.9	788 (10.0%)	9.4–10.7	92 (5.8%)	4.6–6.9	< 0.01
Endocrine comorbidities							
Obesity	1589 (16.8%)	16.1–17.6	1206 (15.4%)	14.6–16.2	383 (24.0%)	21.9–26.1	< 0.01
Thyroid disorder	1619 (17.1%)	16.4–17.9	1268 (16.2%)	15.3–17.0	351 (22.0%)	19.9–24.0	< 0.01
Renal comorbidities							
Renal chronic failure	142 (1.5%)	1.3–1.7	27 (0.3%)	0.2–0.5	115 (7.2%)	5.9–8.5	< 0.01
Recurring urinary tract infection	488 (5.2%)	4.7–5.6	339 (4.3%)	3.9–4.8	149 (9.3%)	7.9–10.8	< 0.01
Neoplasic comorbidities							
Any active neoplasia	479 (5.1%)	4.6–5.5	286 (3.6%)	3.2–4.1	193 (12.1%)	10.5–13.7	< 0.01
Breast	74 (0.8%)	0.6–1.0	45 (0.6%)	0.4–0.7	29 (1.8%)	1.2–2.5	< 0.01
Prostate	66 (0.7%)	0.5–0.9	31 (0.4%)	0.3–0.5	35 (2.2%)	1.4–2.9	< 0.01
Skin	60 (0.6%)	0.5–0.8	36 (0.5%)	0.3–0.6	24 (1.5%)	0.9–2.1	< 0.01
Colorectal	57 (0.6%)	0.4–0.7	35 (0.4%)	0.3–0.6	22 (1.4%)	0.8–1.9	< 0.01
Bladder	35 (0.4%)	0.2–0.5	15 (0.2%)	0.1–0.3	20 (1.3%)	0.8–1.8	< 0.01
Lung	34 (0.4%)	0.2–0.5	19 (0.2%)	0.1–0.4	15 (0.9%)	0.5–1.4	< 0.01
Cervix	18 (0.2%)	0.1–0.3	17(0.2%)	0.1–0.3	1 (0.1%)	0.0–0.2	0.20
Liver–Gastrointestinal–Pancreas	21 (0.2%)	0.1–0.3	11 (0.1%)	0.0–0.2	10 (0.6%)	0.2–1.0	< 0.01
Renal	12 (0.1%)	0.1–0.2	3 (0.0%)	0.0–0.1	9 (0.6%)	0.2–0.9	< 0.01
Endometrial	5 (0.1%)	0.0–0.1	3 (0.0%)	0.0–0.1	2 (0.1%)	0.0–0.3	0.17
Leukaemia	27 (0.3%)	0.2–0.4	15 (0.2%)	0.1–0.3	12 (0.8%)	0.3–1.2	< 0.01
Lymphoma	48 (0.5%)	0.4–0.6	31 (0.4%)	0.3–0.5	17 (1.1%)	0.5–1.5	< 0.01
Other neoplasia	38 (0.4%)	0.3–0.5	30 (0.4%)	0.3–0.5	8 (0.5%)	0.1–0.8	0.50

*CI* Confidence Interval; *HIV* Human Immunodeficiency Virus; *COPD* Chronic Obstructive Pulmonary Disease

Regarding the utilization of healthcare services, the mean number of contacts per year by patients with polypharmacy was 30.3 (SD = 24.9), while for patients without polypharmacy it was 10.5 (SD = 13.4). A total of 1581 (98.9%) individuals with polypharmacy made use of PC services, with an average of 25.9 (SD = 21.3) contacts annually. On the other hand, 6274 (80.0%) patients without polypharmacy were PC users, with a mean of 8.8 (SD = 11.2) visits annually. The most common types of contact among the population with polypharmacy were medical contact, with an annual mean of 25.5 (SD = 21); administrative contact, with a mean of 2.2 (SD = 5.3); and laboratory contact, with a mean of 1.4 (SD = 1.9). Most of the patients with polypharmacy decided to contact in person, with a mean per year of 21.6 (SD = 16.5), followed by home visits, with a mean of 3.0 (SD = 8.6), and telephone consultations, with 1.4 (SD = 1.9). The most contacted professional by individuals with polypharmacy was the physician, reaching an annual mean of 12.4 (SD = 9.3), followed by 9.2 visits/year (SD = 12.9) to the nurse, 0.4 (SD = 2.4) to the physiotherapist, 0.3 (SD = 1.1) to the social worker, 0.03 (SD = 0.29) to the dentist, 0.01 (SD = 0.34) to the paediatrician and 0.01 SD = 0.10) to the midwife. In addition, 1,073 (67.1%) polypharmacy subjects utilized HC services, totalling a mean of 4.4 (SD = 7.2) contacts per year. In contrast, 3,070 (39.1%) individuals without polypharmacy made use of the HC, with a mean of 1.7 (SD = 4.0) annual contacts. The mean use of external consultations by patients with polypharmacy was 3.2 (SD = 4.7), 0.7 (SD = 1.4) for emergencies, 0.3 (SD = 2.5) for day hospital care and 0.2 (SD = 0.6) for hospitalization. Only 16 (1.0%) patients with polypharmacy did not use any PC or HC service during the year studied, in contrast with 1,469 (18.7%) patients without polypharmacy who were nonusers. The global data on the use of PC and HC services and divided by having or not having polypharmacy are shown in Table 3*.*

**Table 3:** Service utilization in patients with chronic diseases and with or without polypharmacy

M [SD]	Total patients with chronic diseases 9443 (100%)	95% CI	Non-polypharmacy patients 7845 (83.1%)	95% CI	Polypharmacy patients 1598 (100%)	95% CI	*p*
Total annual healthcare contacts	13.9 [17.6]	13.5–14.2	10.5 [13.4]	10.2–10.8	30.3 [24.9]	29.1–31.6	< 0.01
Primary care							
N ≥ 1 contact (%)*	7855 (83.2%)	82.4–83.9	6274 (80.0%)	79.1–80.9	1581 (98.9%)	98.4–99.4	< 0.01
Total annual primary care contacts	11.7 [14.9]	11.4–12.0	8.8 [11.2]	8.6–9.1	25.9 [21.3]	24.8–26.9	< 0.01
Contact type							
Medical	11.4 [14.6]	11.1–11.7	8.6 [10.9]	8.3–8.8	25.5 [21.0]	24.5–26.6	< 0.01
Administrative	0.9 [3.3]	0.8–1.0	0.7 [2.7]	0.6–0.7	2.2 [5.3]	1.9–2.5	< 0.01
Laboratory	0.8 [1.3]	0.7–0.8	0.6 [1.1]	0.6–0.7	1.4 [1.9]	1.3–1.4	< 0.01
Mode of contact							
In person	10.7 [12.7]	10.4–11.0	8.5 [10.5]	8.3–8.7	21.6 [16.5]	20.1–22.4	< 0.01
Home visit	0.6 [4.0]	0.6–0.7	0.1 [1.6]	0.1–0.2	3.0 [8.6]	2.6–3.4	< 0.01
Telephone	0.4 [2.3]	0.3–0.4	0.2 [1.1]	0.2–0.2	1.4 [4.8]	1.1–1.6	< 0.01
Professional contacted							
Physician	6.3 [7.1]	6.1–6.4	5.0 [5.8]	5.0–5.2	12.4 [9.3]	11.9–12.9	< 0.01
Nurse	3.2 [7.1]	3.1–3.4	2.0 [4.3]	1.9–2.1	9.2 [12.9]	8.6–9.8	< 0.01
Physiotherapist	0.3 [2.0]	0.3–0.3	0.3 [1.9]	0.2–0.3	0.4 [2.4]	0.3–0.5	0.01
Social worker	0.1 [0.6]	0.1–0.1	0.04 [0.4]	0.0–0.1	0.3 [1.1]	0.2–0.3	< 0.01
Dentist	0.04 [0.4]	0.04–0.05	0.04 [0.4]	0.0–0.1	0.03 [0.29]	0.02–0.05	0.16
Paediatrician	0.01 [0.2]	0.00–0.01	0.00 [0.1]	0.00–0.01	0.01 [0.34]	0.00–0.03	0.24
Midwife	0.1 [0.7]	0.1–0.1	0.1 [0.8]	0.1–0.2	0.01 [0.10]	0.00–0.01	< 0.01
Hospital care							
*N* ≥ 1 contact (%)*	4143 (43.9%)	42.9–44.9	3070 (39.1%)	38.1–40.2	1073 (67.1%)	64.8–69.5	< 0.01
Total annual hospital care contacts	2.2 [4.8]	2.1–2.3	1.7 [4.0]	1.6–1.8	4.4 [7.2]	4.1–4.8	< 0.01
Contact type							
External consultations	1.7 [3.4]	1.6–1.7	1.3 [3.0]	1.3–1.4	3.2 [4.7]	3.0–3.5	< 0.01
Emergency department	0.3 [0.9]	0.3–0.3	0.2 [0.7]	0.2–0.3	0.7 [1.4]	0.7–0.8	< 0.01
Day hospital care	0.1 [1.9]	0.1–0.2	0.1 [1.7]	0.1–0.1	0.3 [2.5]	0.2–0.4	< 0.01
Hospitalization	0.1 [0.3]	0.1–0.1	0.04 [0.2]	0.03–0.04	0.2 [0.6]	0.2–0.2	< 0.01
Non-primary-care, non-hospital-care users							
*N* (%)	1485 (15.7%)	15.0–16.5	1469 (18.7%)	17.9–19.6	16 (1.0%)	0.5–1.5	< 0.01

^*^Measured by total number of patients with at least 1 contact

*M [SD]* Mean [Standard Deviation]; *CI* Confidence Interval

High-risk individuals employed more healthcare services than medium- and low-risk individuals, as depicted by annual averages of 44.6 (SD = 4.3), 30.1 (SD = 20.5), and 20.1 (SD = 16.8), respectively. For PC services, the means per year were 36.2 (SD = 28.4), 26.1 (SD = 18.3), and 17.9 (SD = 15.4), while for HC care, they were 8.4 (SD = 11.3), 4.1 (SD = 5.4), and 2.2 (SD = 4.0). Only 87.5% vs. 69.9% vs. 47.6% of the subjects were HC users. High-risk patients did not turn to the physiotherapist or to the dentist, whereas these two professionals were the third and fifth most visited by medium- and low-risk individuals. High- and medium-risk individuals were more often put in day hospital care, while low-risk patients were hospitalized more often. Among the subjects who did not use PC and HC services, 0.6% were high-risk, 0.9% were medium-risk and 1.5% were low-risk individuals. The mean utilization of healthcare services in a year by patients with polypharmacy, stratified by AMG risk level, is detailed in Table 4*.*

**Table 4:** Mean annual utilization of healthcare services by patients with polypharmacy, according to AMG risk level

M [SD]	High risk 351 (22.0%)	Medium risk 774 (48.4%)	Low risk 473 (29.6%)	*p*
Total annual healthcare contacts	44.6 [34.3]	30.1 [20.5]	20.1 [16.8]	< 0.01
**Primary care**				
N ≥ 1 contact (%)*	348 (99.1%)	767 (99.1%)	466 (98.5%)	0.57
Total annual primary care contacts	36.2 [28.4]	26.1 [18.3]	17.9 [15.4]	< 0.01
Contact type				
Medical	35.9 [28.2]	25.6 [18.0]	17.7 [15.3]	< 0.01
Administrative	3.1 [6.4]	2.1 [5.1]	1.7 [4.5]	< 0.01
Laboratory	1.9 [2.5]	1.3 [1.7]	1.0 [1.6]	< 0.01
Mode of contact				
In person	27.8 [20.1]	22.1 [15.5]	16.1 [13.1]	< 0.01
Home visit	6.1 [14.2]	2.7 [6.7]	1.3 [4.0]	< 0.01
Telephone	2.7 [8.6]	1.3 [3.1]	0.5 [2.2]	< 0.01
Professional contacted				
Physician	16.9 [12.4]	12.7 [8.0]	8.6 [6.5]	< 0.01
Nurse	14.0 [19.0]	9.0 [10.4]	6.0 [9.4]	< 0.01
Physiotherapist	0 [0]	0.7 [3.0]	0.3 [2.1]	< 0.01
Social worker	0.34 [1.6]	0.25 [0.95]	0.18 [0.90]	0.05
Dentist	0 [0]	0.05 [0.37]	0.03 [0.27]	0.05
Paediatrician	0.04 [0.69]	0.01 [0.14]	0.01 [0.10]	0.75
Midwife	0.02 [0.15]	0.01 [0.09]	0.01 [0.08]	0.25
Hospital care				
N ≥ 1 contact (%)*	307 (87.5%)	541 (69.9%)	225 (47.6%)	< 0.01
Total annual hospital care contacts	8.4 [11.3]	4.1 [5.4]	2.2 [4.0]	< 0.01
Contact type				
External consultations	5.6 [6.3]	3.1 [4.2]	1.7 [3.3]	< 0.01
Emergency department	1.3 [1.7]	0.7 [1.3]	0.4 [0.9]	< 0.01
Day hospital care	1.0 [5.1]	0.1 [0.9]	0.0 [0.2]	< 0.01
Hospitalization	0.5 [0.9]	0.1 [0.4]	0.1 [0.3]	< 0.01
Non-primary-care, non-hospital-care users				
*N* (%)	2 (0.6%)	7 (0.9%)	7 (1.5%)	0.40

^*^Measured by total number of patients with at least 1 contact

*M [SD]* Mean [Standard Deviation]; *CI* Confidence Interval

Comparing both sexes, males made greater use of healthcare resources than females, averaging 33.4 (SD = 30.3) vs. 28.9 (SD = 22.0) contacts annually, 27.6 (SD = 24.8) vs. 25.1 (SD = 19.4) to PC and 5.8 (SD = 9.8) vs. 3.8 (SD = 5.6) to HC, with 73.4% vs. 64.3% of them HC users. Patients aged 75 or younger utilized healthcare services more than the elderly, with a mean of 37.0 (SD = 29.0) vs. 30.0 (SD = 24.3) visits per year, 28.0 (SD = 22.7) vs. 25.7 (SD = 21.1) being to PC and 8.8 (SD = 12.7) vs. 4.0 (SD = 6.2) being to HC, where 79.4% vs. 66.9% were HC users and 0.6% vs. 1.0% were not PC and HC users. Younger patients were admitted for day hospital care more than for emergencies; in contrast, older individuals were more likely to go to the emergency department. Table 5 presents the mean utilization of healthcare services by patients with polypharmacy in a year, according to sex and age group.

**Table 5:** Mean annual utilization of healthcare services by patients with polypharmacy, by sex and age group

M [SD]	Female 1101 (68.9%)	Male 497 (31.1%)	*p*	Age ≤ 75 155 (9.7%)	Age > 75 1443 (90.3%)	*p*
Total annual healthcare contacts	28.9 [22.0]	33.4 [30.3]	0.01	37.0 [29.0]	30.0 [24.3]	< 0.01
Primary care						
N ≥ 1 contact (%)*	1090 (99.0%)	491 (98.8%)	0.71	154 (99.4%)	1,427 (98.9%)	0.59
Total annual primary care contacts	25.1 [19.4]	27.6 [24.8]	0.18	28.0 [22.7]	25.7 [21.1]	0.43
Contact type						
Medical	24.7 [19.1]	27.4 [24.8]	0.12	27.4 [22.2]	25.3 [20.9]	0.47
Administrative	1.9 [5.0]	2.8 [5.9]	0.03	2.0 [5.2]	2.2 [5.3]	0.81
Laboratory	1.4 [1.9]	1.2 [1.8]	0.04	1.5 [1.9]	1.3 [1.9]	0.13
Mode of contact						
In person	20.8 [15.1]	23.4 [19.1]	0.04	25.2 [21.1]	21.25 [15.9]	0.11
Home visit	2.9 [7.0]	3.2 [11.3]	0.07	1.6 [4.9]	3.2 [8.8]	< 0.01
Telephone	1.4 [5.3]	1.2 [3.3]	0.78	1.2 [5.0]	1.4 [4.8]	0.01
Professional contacted						
Physician	12.4 [9.7]	12.4 [8.3]	0.55	13.7 [10.4]	12.3 [9.1]	0.34
Nurse	8.6 [9.9]	10.6 [17.7]	0.53	10.2 [14.2]	9.1 [12.7]	0.93
Physiotherapist	0.4 [2.5]	0.4 [2.2]	0.79	0.5 [2.7]	0.4 [2.4]	0.80
Social worker	0.3 [1.2]	0.2 [0.9]	0.10	0.1 [0.4]	0.3 [1.2]	0.09
Dentist	0.03 [0.30]	0.03 [0.28]	0.80	0.02 [0.18]	0.03 [0.30]	0.97
Paediatrician	0.02 [0.41]	0.01 [0.11]	0.50	0 [0]	0.02 [0.36]	0.39
Midwife	0.01 [0.12]	0.00 [0.05]	0.05	0.03 [0.20]	0.01 [0.09]	0.14
Hospital care						
*N* ≥ 1 contact (%)*	708 (64.3%)	365 (73.4%)	< 0.01	123 (79.4%)	965 (66.9%)	< 0.01
Total annual hospital care contacts	3.8 [5.6]	5.8 [9.8]	< 0.01	8.8 [12.7]	4.0 [6.2]	< 0.01
Contact type						
External consultations	2.7 [4.1]	4.2 [5.7]	< 0.01	6.1 [6.8]	3.0 [4.4]	< 0.01
Emergency department	0.7 [1.3]	0.8 [1.5]	0.30	1.0 [1.6]	0.7 [1.3]	< 0.01
Day hospital care	0.2 [1.4]	0.6 [4.0]	0.20	1.4 [6.2]	0.2 [1.6]	< 0.01
Hospitalization	0.2 [0.5]	0.3 [0.7]	0.15	0.3 [0.8]	0.2 [0.5]	0.06
Non-primary-care, non-hospital-care users						
*N* (%)	11 (1.0%)	5 (1.0%)	0.99	1 (0.6%)	15 (1.0%)	0.64

^*^Measured by total number of patients with at least 1 contact

*M [SD]* Mean [Standard Deviation]; *CI* Confidence Interval

The negative binomial analysis revealed that the clinical variables associated with a greater use of PC services by polypharmacy patients were an increased complexity index (*Z* = 2.93, *p* < 0.01), a high risk level (*Z* = 2.29, *p* = 0.02) and dysrhythmias (*Z* = 2.65, *p* < 0.01). Regarding the HC services, we found that males tended to utilize these services more (*Z* = 2.06, *p* = 0.04), unlike the total Spanish (*Z* = − 7.32, *p* < 0.01) and older patients (*Z* = − 5.86, *p* < 0.01), who made lesser use of them. The clinical factors linked with a higher utilization of HC services were also an elevated complexity index (*Z* = 4.61, *p* < 0.01) and a high risk level (*Z* = 4.26, *p* < 0.01), whereas the functional characteristics associated were being in palliative care (*Z* = 2.48, *p* = 0.01) and having a primary caregiver (*Z* = 2.12, *p* = 0.03). In contrast, being immobilized at home was linked with a lower use of HC services (Z = − 2.5; p = 0.01). In addition, active neoplasia (*Z* = 2.67, *p* < 0.01), more specifically lymphoma (*Z* = 3.19, *p* < 0.01) and leukaemia (*Z* = 2.23, *p* = 0.03), together with arthritis (*Z* = 2.18, *p* = 0.03), made these patients more likely to use HC resources, while those undergoing recurring urinary tract infection showed the opposite tendency (*Z* = − 2.8, *p* < 0.01). All the factors we found associated with the utilization of PC and HC services in polypharmacy patients are described in Table 6.

**Table 6:** Factors associated with the utilization of primary and hospital care services by patients with polypharmacy

Variables	Coefficient	SE	95% CI	*Z* value	*P* >|z|
Primary care users					
Complexity index	0.17	0.06	0.06–0.29	2.93	< 0.01
Dysrhythmias	0.12	0.05	0.03–0.22	2.65	< 0.01
High risk level by AMG	0.11	0.05	0.02–0.20	2.29	0.02
Hospital care users					
Complexity index	0.29	0.06	0.17–0.41	4.61	< 0.01
High risk level by AMG	0.22	0.05	0.12–0.32	4.26	< 0.01
Active neoplasia	0.13	0.05	0.04–0.23	2.67	< 0.01
Primary caregiver	0.12	0.06	0.01–0.22	2.12	0.03
Lymphoma	0.09	0.03	0.04–0.15	3.19	< 0.01
Male sex	0.07	0.04	0.00–0.14	2.06	0.04
Palliative care	0.07	0.03	0.02–0.13	2.48	0.01
Arthritis	0.07	0.03	0.01–0.14	2.18	0.03
Leukaemia	0.06	0.03	0.01–0.11	2.23	0.03
Recurring urinary tract infection	− 0.09	0.03	(− 0.15)–(− 0.03)	− 2.8	< 0.01
Immobilized	− 0.14	0.06	(− 0.25)–(− 0.03)	− 2.5	0.01
Age	− 0.20	0.03	(− 0.26)–(− 0.13)	− 5.86	< 0.01
Spanish origin	− 0.24	0.03	(− 0.30)–(− 0.17)	− 7.32	< 0.01

*CI* Confidence Interval; *SE* Standard Error

## Discussion

In the area studied, 61.3% patients had chronic diseases, of which 16.9% had polypharmacy and 83.1% were patients without polypharmacy. This polypharmacy population over 18 years of age defined by the usual intake of 6 or more medications, when compared to the non-polypharmacy patients, showed a higher proportion of women and individuals at high risk, with a more advanced age and greater disease complexity, and suffering from a larger number of comorbidities, in addition to requiring far more assistance and care. As a consequence of their complex condition, subjects with polypharmacy made substantially greater use of PC and HC services. The most significant factors associated with a frequent use of PC services by polypharmacy patients were a high complexity index, having a primary caregiver, and suffering from dysrhythmia and active neoplasia; for HC services, the most significant factors were being over 75 years, being in palliative care, an elevated complexity index and the comorbidities leukaemia, lymphoma, lung neoplasia, active neoplasia and chronic obstructive pulmonary disease.

Our prevalence of polypharmacy fits into the wide range, from 4 to 96.5%, described in a narrative review from 2021 [3]. Still, our estimation may be considered relatively low, as only patients who concomitantly took six or more medications were considered [10], while the most common definitions refer to five or more medications [3]. The same happens regarding age, as many studies are done exclusively in the elderly [5], who have higher frequencies of polypharmacy than populations that also include younger age groups, as ours did. In addition, our low prevalence can be explained, because other studies focus only on specific populations or healthcare settings, such as patients frequently admitted to hospital or registered in acute care units, which usually use more medications [3]. For instance, a Spanish study that analysed at primary care level all participants aged over 14 taking at least 5 medications calculated for the same year of our study a prevalence quite similar to ours[4]. Therefore, polypharmacy studies should be compared with caution, paying special attention to the definition employed, the age range covered and the specific population or healthcare setting studied.

The mean age of our population with polypharmacy was 82.7 years, close to the upper limit of the range of 26–87 years reported in a recent meta-analysis [5], as most of our polypharmacy population was older than 75. Polypharmacy patients showed a much higher mean age but also more impaired functional capacity and a higher prevalence of women than the non-polypharmacy ones, which is explained by the association of polypharmacy with age, the elderly age group having the poorest physical function and women having the longest lifespan [3, 5, 12–22].

In comparison with the patients without polypharmacy, almost all patients with polypharmacy had multimorbidity, and they had more chronic diseases on average, in line with other polypharmacy populations [16–18] although reporting different mean numbers of comorbidities owing to the lack of consensus to define and measure multimorbidity, as well as the wide variation in the diseases defined as chronic and discrepancies in the length of time a condition must be present to be defined as chronic [23]. Nevertheless, published studies agreed that the number of chronic diseases rises in parallel with the number of prescribed medications, sometimes analogous to the emergence of excessive polypharmacy [12–14, 16–19, 21, 22, 24–26].

The most prevalent comorbidities within the polypharmacy population were hypertension, dyslipidaemia, diabetes, osteoporosis, dysrhythmias, arthrosis and obesity, in accordance with other populations with polypharmacy [13, 19, 27] and with the elderly population with polypharmacy [12, 15, 18, 21, 24–26, 28]. Our results agree with earlier findings that cardiovascular diseases are more prevalent in men, while arthritis, osteoporosis and thyroid disorder are more prevalent in women [12, 15, 16, 18, 26].

High- and medium-risk patients were more prevalent among polypharmacy patients than among those without polypharmacy, as well as when contrasting to the overall population of Madrid or other regions of Spain stratified by the AMG [20, 29], although similar to another Spanish study with adults over 65 years living alone [30]. Younger patients and males showed higher levels of risk and complexity, probably because their severe conditions shortened their life expectancy and men have a shorter lifespan [21]. High-risk individuals generally presented increased ratios of functional impairment, consistent with previous studies [17]; however, the demand for a primary caregiver grew with the reduction in risk level, since the lack of this service could contribute to the increase in risk level, as observed in other studies [31].

### Use of primary health care services

Patients with polypharmacy made great use of PC services, accounting for a mean of 25.9 contacts annually, similar in number to that observed within other polypharmacy populations [32, 33], but lower than that in other studies that defined polypharmacy as the use of 5 or more medications [21, 27]. The frequency of contacts with PC described for polypharmacy patients tripled that registered by non-polypharmacy patients, and the group of polypharmacy high risk patients registered the highest mean of contacts with PC, pursuant to the fact that the use of PC increases with the number of medicines consumed [12, 21, 22, 25, 34]. Almost all patients with polypharmacy used PC services, while a lower percentage of non-polypharmacy patients were PC users, in line with other studies of patients with chronic diseases and with or without polypharmacy [14, 28, 30, 35, 36]. The most common type of contact was medical contact in person, in agreement with other studies performed in the Community of Madrid involving patients with chronic diseases and geriatric patients [35, 36]. The mean numbers of telephone contacts and home visits were expected to be higher due to the high prevalence of functional impairment in this predominantly older population [37], nevertheless, at present telephone contacts are assumed to be higher now, since the COVID-19 pandemic occurred after the data collection of this study.

The professional most frequently contacted was the physician, in line with previous studies [34, 38], explained by the fact that Spain, like many other countries, follows a gatekeeping approach, where the general practitioner is the first point of contact with the healthcare system and who, if deemed appropriate, refers the patient to other professionals [10]. The average number of contacts that patients with polypharmacy had with the general practitioner doubled the mean for the overall population in Madrid in 2015 [29]. The second most visited medical provider was the nurse, also in line with other studies [34, 38]. Although the mean nurse attendance was lower than that with the physician, it was higher than in other studies [28, 34] and close to the physician average, following the Madrid care programme for the elderly with polypharmacy, where the nurse plays an important role, in coordination with the physician, in controlling polypharmacy and trying to reduce it through deprescription [39]. For the same year when our study was conducted and in comparison with the total population of Madrid, the number of contacts with the nurse was quadrupled in the polypharmacy population [29]. Attending to Madrid polypharmacy programme, social workers should also assume a significant function in the management of these patients [39], but contact with these professionals was still very low in our population.

Unlike other studies [26, 30, 34], we could not find many statistically significant differences regarding the use of PC services between sexes or between age groups younger and older than 75 years. The most remarkable difference was that the elderly made more telephone consultations and received more home visits because of their more incapacitating conditions. We did find differences in the overall use of healthcare resources concerning PC and HC together. Males younger than 75 years were the ones who made a greater use of services, as they were the ones in our population with higher risk levels and complexity indexes, because men usually suffer more from severe and life-threatening illnesses, meaning they must make use of healthcare services and resources more often and they have a shorter life expectancy [25, 40]. Our results are clearly opposed to the greater proportion of contacts with the healthcare system by female and elderly people reported in other studies [26, 30]. This might be because these studies mainly reported unadjusted numbers of healthcare visits, and when adjusting for individuals with similar levels of chronic conditions, functional limitations, and disability, women have fewer healthcare contacts than males [41], supporting our results.

The factors significantly associated with a higher number of contacts with PC by polypharmacy patients were a high level of risk and an elevated weight of the complexity index, since these patients presented with more comorbidities usually requiring further needs for care, which generates higher healthcare expenditures [12, 20, 21, 30]. The prescription of medicines for these patients should be done with caution, since, as consequence of their complex condition, they may visit multiple medical specialists, who need to carefully communicate with the patient as well as with other physicians and prescribers to be aware of all the medications they are taking and to avoid inappropriate prescribing [5, 32, 39, 42]. We also found that dysrhythmia was linked to a higher use of PC services, given that this disease requires continuous monitoring by PC, involving frequent antiarrhythmic and anticoagulant dose adjustments and regular symptom control and risk factor management [43].

### Use of hospital health care services

Patients with polypharmacy also made a notably higher use of HC services than chronic and non-polypharmacy individuals, according to the widely reported strong association between the intake of multiple drugs and greater HC utilization, in many cases triggered by adverse drug reactions [3, 5, 13, 14, 21, 24]. The number of emergency department visits in our polypharmacy population was lower than that of most studies [33, 42], but in other cases similar [13] or higher [25, 32, 44]. These differences could be explained by the distinct populations analysed and the definition of polypharmacy considered. Our lower numbers could point to a more appropriate and safe use of drugs by these patients and a more suitable prescription of medications by the healthcare professionals in the Community of Madrid [42]. In a similar way, the rates of hospitalizations were lower than those observed in many previous reviews [13, 27, 42, 44], although similar [32] or lower [25] to others. In comparison with the total population of the Community of Madrid, patients with polypharmacy visited the emergency room and were hospitalized around two times more [29], coinciding with the results reported by Nascimento et al. [13]. As expected and pursuant to other studies [12, 21], polypharmacy patients at higher risk made a higher use of HC services, the high-risk patients four times as much as the low-risk patients and two times as much as those with medium risk. Again, males under 75 years were the group who utilized HC services the most due to their more complex conditions, which is in line with previous findings [26].

The statistically significant variables associated with a higher use of HC resources were an increased complexity index and a high AMG risk level, which were also, along with being male, the factors associated with the increased use of PC services, in line with what was discussed above. Older age showed a negative correlation with the use of HC services, contrary to most studies that found a strong positive association [45, 46]. This could be because old age is the age group with the highest rates of immobilization, making for a great struggle for them to come to the hospital [45], while they can receive PC family doctor home visits. Having a primary caregiver was associated with a higher use of HC services, as previously shown, since primary caregivers help and inform the patient about healthcare options and provide constant care and support to the patient [47]. Polypharmacy patients in palliative care also showed a stronger tendency to visit HC facilities, because these services need to be periodically delivered in specific palliative care units located in hospitals [29]. The results indicated a higher trend of foreigners visiting the HC facilities, explained by a miscommunication with healthcare providers due to language differences and misunderstanding of the treatment plans and instructions, leading to an overuse of healthcare services, as described previously [14]. Besides, polypharmacy patients with active neoplasia were more likely to use HC resources given that cancer is one of the main reasons for hospital attendance in Spain, and most of the treatments are carried out at hospital level of care [29]. In addition, treatment with antineoplastic agents presents toxicities that increase the number of visits to the emergency department and hospitalizations [48]. Arthritis was also linked to a higher utilization of HC services as this musculoskeletal disease has a significant impact on the quality of life and functional capacity of those affected, resulting in a sizable impact on the healthcare system [49].

### Limitations and strengths

The use of a cross-sectional study design means that the associations identified between the variables studied should be interpreted cautiously. In addition, the extraction of information from the digital medical record system could lead to heterogeneity given the variability in the registration of the information between healthcare professionals, in addition to the different definition of polypharmacy they considered and their individual prescribing preferences, which may lead to errors in the calculation of the polypharmacy prevalence within our population. Nevertheless, the utilization of this kind of clinical-administrative registry for epidemiological studies provides data from a global approach covering almost all individuals and not of partial samples, reducing selection and memory biases. Patients with private insurance may not be represented in the total population studied, although this is indeed unlikely to deeply influence the results, since more than 90% of the inhabitants of Madrid have public health insurance [39].

Although the data were extracted from a unique healthcare area, all the results are representative of the Madrid population with chronic diseases and polypharmacy and can be extrapolated to the rest of the Spanish territory, since this health area serves a widely heterogeneous and varied group of people, including patients of different nationalities, in addition to the fact that the patients from this basic health area have shown similar trends to the ones observed outside the studied area.

Morbidity grouping algorithms have commonly aroused doubts on the criteria and procedures followed to calculate the complexity and do not consider socioeconomic status, disability, fragility, the need for care, clinical parameters or severity assessment scales [7]. In this context, the AMG tool stands out for overcoming these limitations and has proven its validity compared to other stratification tools. Some chronic diseases have not been considered, because the AMG grouper only contemplates a series of specific ones defined in Additional file 1: Table S1 [8]; therefore, the prevalence calculated here could be undervalued, since we identified only the polypharmacy individuals out of the chronic population we identified at first.

### Implications

The rise in the prevalence of polypharmacy as a result of the global ageing population is one of the greatest challenges faced by healthcare systems worldwide. Polypharmacy patients use an extremely high proportion of PC and HC services, generating significant healthcare expenses. These patients require an individual case management approach based on personalized care and treatment plans established according to their singular health conditions. In this context, the Community of Madrid implemented a care programme for patients with polypharmacy, as well as a specific care programme for chronic complex patients, fostering the coordination and continued action by all health professionals involved in patient care and the implementation of systematic revision of health status, treatments, health education and the use of means to support clinical follow-up and therapeutic compliance adapted to each particular patient [39].

The present study is the first to provide data on chronic patients with polypharmacy according to their AMG risk level, in addition to providing valuable information about the utilization of healthcare services and needs of care. The comprehensive understanding and interpretation of all this information will assist in the coordination between the primary and the hospital level of care and will help professionals optimize the management, treatment and rational use of medicines of polypharmacy patients, making it possible to reduce the extreme healthcare expenses originated by these patients.

## Conclusions

Patients with polypharmacy represented a considerable percentage of the total number of patients with chronic diseases. In comparison with non-polypharmacy patients, the polypharmacy population was older, with a greater predominance of women and characterized by important needs for care, with higher rates of multimorbidity and suffering from a larger number of chronic diseases, and this situation was worsened with the aggravation of the level of risk and complexity. Due to their condition, polypharmacy patients had a higher utilization rate of PC services, principally directing their visits to the general practitioner or nurse, and their use of HC resources was elevated as well, mainly in the form of external consultations. This increased utilization of healthcare services was associated with a high risk level and complexity index and other clinical factors, such as active neoplasia, as well as functional factors, such as the need for a primary caregiver. These findings could help policy makers in the allocation of healthcare services with the purpose of improving the management of polypharmacy and the rational use of medicines, as well as for reducing the costs associated with the extensive use of services by highly medication-consuming population.

### Supplementary Information

**Additional file 1: Table S1.** Types of chronic diseases considered by the Adjusted Morbidity Group (AMG) in the Community of Madrid. **Table S2.** Sociodemographic, functional and clinical characteristics of the patients with polypharmacy by AMG risk level. **Table S3.** Sociodemographic, functional and clinical characteristics of the polypharmacy patients by sex and age groups. **Table S4.** Comorbidities in patients with polypharmacy by AMG risk level. **Table 5.** Comorbidities in patients with polypharmacy by sex and age groups.

## Data Availability

The data sets generated and analysed during the study are not publicly available, because they belong to Madrid Health Service, but they are available from the corresponding author upon reasonable request.

## Abbreviations

AMG: Adjusted morbidity groups
COPD: Chronic obstructive pulmonary disease
HC: Hospital care
HIV: Human immunodeficiency virus
PC: Primary care
WHO: World Health Organization
