# Supplementary material for: Differences in healthcare service utilization in patients with polypharmacy according to their risk level by adjusted morbidity groups: a population-based cross-sectional study
Source: J Pharm Policy Pract. 2023 Nov 28;16:161. doi: 10.1186/s40545-023-00665-7 (PMC10683272; doi:10.1186/s40545-023-00665-7)
Supplement: Supplementary file 1 — Additional file 1: Table S1. Types of chronic diseases considered by the Adjusted Morbidity Group (AMG) in the Community of Madrid. Table S2. Sociodemographic, functional and clinical characteristics of the patients with polypharmacy by AMG risk level. Table S3. Sociodemographic, functional and clinical characteristics of the polypharmacy patients by sex and age groups. Table S4. Comorbidities in patients with polypharmacy by AMG risk level. Table 5. Comorbidities in patients with polypharmacy by sex and age groups. [file 40545_2023_665_MOESM1_ESM.docx]

| **Table S1. Types of chronic diseases considered by the Adjusted Morbidity Group (AMG) in the Community of Madrid.** |
| --- |
| Alcoholism |
| Anaemia |
| Aorta aneurysm |
| Anxiety |
| Arthritis |
| Arthrosis |
| Asthma |
| Attention-Deficit/Hyperactivity Disorder (ADHD) |
| Bladder cancer |
| Breast cancer |
| Cardiopulmonary disease |
| Central nervous system cancer |
| Cervical cancer |
| Cirrhosis |
| Colon cancer |
| Dementia |
| Depression |
| Diabetes Mellitus |
| Dyslipidaemia |
| Dysrhythmias |
| Ear, nose and throat cancer |
| Endometrial cancer |
| Epilepsy |
| Gastrointestinal ulcer |
| Glaucoma |
| Heart chronic failure |
| Hepatoblastoma |
| Hodgkin/Other lymphomas |
| Human Immunodeficiency Virus (HIV) |
| Dyslipidaemia |
| Hypertension |
| Ischemic heart disease |
| Leukaemia |
| Liver cancer |
| Lung cancer |
| Mental retardation |
| Multiple sclerosis |
| Obesity |
| Chronic Obstructive Pulmonary Disease (COPD) |
| Osteoarthritis |
| Osteoporosis |
| Pancreatic cancer |
| Parkinson |
| Prostate cancer |
| Renal cancer |
| Renal chronic failure |
| Retinoblastoma |
| Schizophrenia |
| Skin cancer |
| Soft tissues cancer |
| Stomach cancer |
| Stroke |
| Substance abuse |
| Testicle cancer |
| Thyroid cancer |
| Thyroid disorder |
| Ulcerative colitis |
| Valvular heart disease |
| Vasculitis |

| **Table S2. Sociodemographic, functional and clinical characteristics of the patients with polypharmacy by AMG risk level**. | | | | |
| --- | --- | --- | --- | --- |
| n (%) | **High risk**  351 (22.0%) | **Medium risk**  774 (48.4%) | **Low risk**  473 (29.6%) | **p** |
| **Sociodemographic variables** | | | | |
| Female | 191 (54.4%) | 541 (69.9%) | 369 (78.0%) | <0.01 |
| Age* | 79.9 [10.8] | 83.5 [5.3] | 83.5 [5.6] | <0.01 |
| ≤ 75 years  > 75 years | 90 (25.6%)  261 (74.4%) | 41 (5.3%)  733 (94.7%) | 24 (5.1%)  449 (94.9%) | <0.01 |
| Origin Spain  Rest of Europe  Rest of the world | 311 (88.6%)  6 (1.7%)  34 (9.7%) | 685 (88.5%)  24 (3.1%)  65 (8.4%) | 385 (81.4%)  16 (3.4%)  72 (15.2%) | <0.01 |
| **Functional variables** | | | | |
| Immobilized | 111 (31.6%) | 111 (14.3%) | 35 (7.4%) | <0.01 |
| Institutionalized | 34 (9.7%) | 43 (5.6%) | 69 (8.2%) | 0.03 |
| Primary caregiver | 93 (3.8%) | 89 (11.5%) | 18 (26.5%) | <0.01 |
| Home support | 26 (7.4%) | 36 (4.7%) | 9 (1.9%) | <0.01 |
| Palliative care | 26 (7.4%) | 3 (0.4%) | 1 (0.2%) | <0.01 |
| **Clinical variables** | | | | |
| Complexity index* | 31.2 [12.5] | 13.0 [2.7] | 6.6 [1.6] | <0.01 |
| Chronic diseases* | 7.3 [2.2] | 4.6 [1.5] | 3.0 [1.2] | <0.01 |
| Multimorbidity | 351 (100.0%) | 759 (98.1%) | 418 (88.4%) | <0.01 |
| * Mean [standard deviation].  Abbreviations: AMG, Adjusted Morbidity Groups. | | | | |

| **Table S3. Sociodemographic, functional and clinical characteristics of the polypharmacy patients by sex and age groups.** | | | | | | |
| --- | --- | --- | --- | --- | --- | --- |
| n (%) | **Female**  1,101 (68.9%) | **Male**  497 (31.1%) | **p** | **Age ≤ 75**  155 (9.7%) | **Age > 75**  1,443 (90.3%) | **p** |
| **Sociodemographic variables** | | | | | | |
| Female | - | - | - | 84 (54.2%) | 1,017 (70.5%) | <0.01 |
| Age* | 83.3 [6.8] | 81.4 [7.6] | <0.01 | 69.2 [8.8] | 84.2 [5.1] | <0.01 |
| ≤ 75 years  > 75 years | 84 (7.6%)  1,017 (92.4%) | 71 (14.3%)  426 (85.7%) | <0.01 | -  - | -  - | - |
| Origin Spain  Rest of Europe  Rest of the world | 949 (86.2%)  34 (3.1%)  118 (10.7%) | 432 (86.9%)  12 (2.4%)  53 (10.7%) | 0.76 | 134 (86.5%)  3 (1.9%)  18 (11.6%) | 1,247 (86.4%)  43 (3%)  153 (10.6%) | 0.72 |
| **Functional variables** | | | | | | |
| Immobilized | 193 (17.5%) | 64 (12.9%) | 0.02 | 11 (7.1%) | 246 (17%) | <0.01 |
| Institutionalized | 91 (8.3%) | 25 (5.0%) | 0.02 | 2 (1.3%) | 114 (7.9%) | <0.01 |
| Primary caregiver | 146 (13.3%) | 54 (10.9%) | 0.18 | 13 (8.4%) | 187 (13.0%) | 0.10 |
| Home support | 51 (4.6%) | 20 (4.0%) | 0.59 | 4 (2.6%) | 67 (4.6%) | 0.24 |
| Palliative care | 12 (1.1%) | 18 (3.6%) | <0.01 | 7 (4.5%) | 23 (1.6%) | 0.01 |
| **Clinical variables** | | | | | | |
| AMG risk level High  Medium  Low | 191 (17.3%)  541 (49.1%)  369 (33.5%) | 160 (32.2%)  233 (46.9%)  104 (20.9%) | <0.01 | 90 (58.1%)  41 (26.5%)  24 (15.5%) | 261 (18.1%)  733 (50.8%)  449 (31.1%) | <0.01 |
| Complexity index* | 13.9 [10.0] | 17.8 [8.0] | <0.01 | 21.9 [13.3] | 14.4 [10.4] | <0.01 |
| Chronic diseases* | 4.7 [2.2] | 4.8 [2.3] | 0.84 | 6.0 [2.6] | 4.6 [2.1] | <0.01 |
| Multimorbidity | 1,059 (96.2%) | 469 (94.4%) | 0.10 | 152 (98.1%) | 1,376 (95.4%) | 0.12 |
| * Mean [standard deviation].  Abbreviations: AMG, Adjusted Morbidity Groups. | | | | | | |

| **Table S4.**  **Comorbidities in patients with polypharmacy by AMG risk level.** | | | | | |
| --- | --- | --- | --- | --- | --- |
| n (%) | | **High risk**  351 (22.0%) | **Medium risk**  774 (48.4%) | **Low risk**  473 (29.6%) | **p** |
| ***Haematic*** ***comorbidities*** | Anaemia | 92 (26,2%) | 74 (9,6%) | 26 (5,5%) | <0.01 |
|  | HIV | 2 (0,6%) | 0 (0,0%) | 0 (0,0%) | 0.03 |
| ***Digestive comorbidities*** | Cirrhosis | 40 (11,4%) | 59 (7,6%) | 12 (2,5%) | <0.01 |
|  | Inflammatory bowel disease | 4 (1.1%) | 8 (1.0%) | 0 (0,0%) | 0.08 |
|  | Gastrointestinal ulcer | 17 (4,8%) | 18 (2,3%) | 12 (2,5%) | 0.06 |
|  | Chronic pancreatitis | 2 (0,6%) | 1 (0,1%) | 0 (0,0%) | 0.15 |
| ***Ocular*** ***comorbidities*** | Glaucoma | 39 (11,1%) | 85 (11,0%) | 42 (8,9%) | 0.44 |
| ***Cardiovascular comorbidities*** | Hypertension | 316 (90,0%) | 629 (81,3%) | 340 (71,9%) | <0.01 |
|  | Diabetes Mellitus | 170 (48,4%) | 226 (29,2%) | 67 (14,2%) | <0.01 |
|  | Dyslipidaemia | 255 (72,6%) | 518 (66,9%) | 250 (52,9%) | <0.01 |
|  | Dysrhythmias | 169 (48,1%) | 181 (23,4%) | 46 (9,7%) | <0.01 |
|  | Heart chronic failure | 115 (32,8%) | 73 (9,4%) | 6 (1,3%) | <0.01 |
|  | Ischaemic heart disease | 103 (29,3%) | 96 (12,4%) | 18 (3,8%) | <0.01 |
|  | Valvular heart disease | 75 (21,4%) | 22 (2,8%) | 3 (0,6%) | <0.01 |
| ***Musculoskeletal comorbidities*** | Arthrosis | 93 (26,5%) | 214 (27,6%) | 79 (16,7%) | <0.01 |
|  | Osteoporosis | 86 (24,5%) | 215 (27,8%) | 105 (22,2%) | 0.08 |
|  | Arthritis | 20 (5,7%) | 34 (4,4%) | 7 (1,5%) | <0.01 |
|  | Lupus | 3 (0,9%) | 0 (0,0%) | 0 (0,0%) | <0.01 |
|  | Vasculitis | 6 (1,7%) | 4 (0,5%) | 2 (0,4%) | 0.06 |
| ***Neurological comorbidities*** | Dementia | 47 (13,4%) | 70 (9,0%) | 32 (6,8%) | <0.01 |
|  | Stroke | 79 (22,5%) | 60 (7,8%) | 16 (3,4%) | <0.01 |
|  | Parkinson | 15 (4,3%) | 31 (4,0%) | 6 (1,3%) | 0.01 |
|  | Epilepsy | 20 (5,7%) | 20 (2,6%) | 8 (1,7%) | <0.01 |
|  | Multiple sclerosis | 0 (0,0%) | 0 (0,0%) | 1 (0,2%) | 0.30 |
| ***Psychiatric comorbidities*** | Alcohol abuse | 33 (9,4%) | 17 (2,2%) | 4 (0,8%) | <0.01 |
|  | Substance abuse | 5 (1,4%) | 1 (0,1%) | 0 (0,0%) | <0.01 |
|  | Anxiety | 66 (18,8%) | 154 (19,9%) | 71 (15,0%) | 0.09 |
|  | Depression | 91 (25,9%) | 150 (19,4%) | 70 (14,8%) | <0.01 |
|  | Bipolar disorder | 4 (1,1%) | 1 (0,1%) | 5 (1,1%) | 0.05 |
|  | Psychotic disorder | 5 (1,4%) | 4 (0,5%) | 6 (1,3%) | 0.23 |
| ***Respiratory comorbidities*** | COPD | 96 (27,4%) | 78 (10,1%) | 15 (3,2%) | <0.01 |
|  | Asthma | 21 (6,0%) | 57 (7,4%) | 14 (3,0%) | <0.01 |
| ***Endocrine comorbidities*** | Obesity | 119 (33,9%) | 194 (25,1%) | 70 (14,8%) | <0.01 |
|  | Thyroid disorder | 88 (25,1%) | 172 (22,2%) | 91 (19,2%) | 0.13 |
| ***Renal comorbidities*** | Renal chronic failure | 88 (25,1%) | 25 (3,2%) | 2 (0,4%) | <0.01 |
|  | Recurring urinary tract infection | 71 (20,2%) | 58 (7,5%) | 20 (4,2%) | <0.01 |
| ***Neoplastic comorbidities*** | Any active neoplasia | 117 (33.3%) | 67 (8.7%) | 9 (1.9%) | <0.01 |
|  | Breast | 18 (5,1%) | 10 (1,3%) | 1 (0,2%) | <0.01 |
|  | Prostate | 21 (6,0%) | 13 (1,7%) | 1 (0,2%) | <0.01 |
|  | Skin | 11 (3,1%) | 11 (1,4%) | 2 (0,4%) | <0.01 |
|  | Colorectal | 12 (3,4%) | 9 (1,2%) | 1 (0,2%) | <0.01 |
|  | Bladder | 15 (4,3%) | 4 (0,5%) | 1 (0,2%) | <0.01 |
|  | Lung | 13 (3,7%) | 1 (0,1%) | 1 (0,2%) | <0.01 |
|  | Cervix | 0 (0,0%) | 1 (0.1%) | 0 (0,0%) | 0.59 |
|  | Liver-Gastrointestinal-Pancreas | 7 (2.0%) | 3 (0.4%) | 0 (0,0%) | <0.01 |
|  | Renal | 7 (2,0%) | 2 (0,3%) | 0 (0,0%) | <0.01 |
|  | Endometrial | 2 (0.6%) | 0 (0,0%) | 0 (0,0%) | 0.03 |
|  | Leukaemia | 8 (2,3%) | 4 (0,5%) | 0 (0,0%) | <0.01 |
|  | Lymphoma | 8 (2,3%) | 7 (0,9%) | 2 (0,4%) | 0.03 |
|  | Other neoplasia | 6 (1.7%) | 2 (0.3%) | 0 (0,0%) | <0.01 |
| Abbreviations: HIV, Human Immunodeficiency Virus; COPD, Chronic Obstructive Pulmonary Disease. | | | | | |

| **Table S5. Comorbidities in patients with polypharmacy by sex and age groups.** | | | | | | | |
| --- | --- | --- | --- | --- | --- | --- | --- |
| n (%) | | **Female**  1,101 (68.9%) | **Male**  497 (31.1%) | **p** | **Age ≤ 75**  155 (9.7%) | **Age > 75**  1,443 (90.3%) | **p** |
| ***Haematic*** ***comorbidities*** | Anaemia | 137 (12.4%) | 55 (11.1%) | 0.43 | 14 (9.0%) | 178 (12.3%) | 0.23 |
|  | HIV | 1 (0.1%) | 1 (0.2%) | 0.56 | 2 (1.3%) | 0 (0%) | <0.01 |
| ***Digestive comorbidities*** | Cirrhosis | 80 (7.3%) | 31 (6.2%) | 0.45 | 27 (17.4%) | 84 (5.8%) | <0.01 |
|  | Inflammatory bowel disease | 7 (0.6%) | 5 (1.0%) | 0.43 | 1 (0.6%) | 11 (0.8%) | 0.87 |
|  | Gastrointestinal ulcer | 24 (2.2%) | 23 (4.6%) | <0.01 | 5 (3.2%) | 42 (2.9%) | 0.83 |
|  | Chronic pancreatitis | 2 (0.2%) | 1 (0.2%) | 0.93 | 1 (0.6%) | 2 (0.1%) | 0.17 |
| ***Ocular*** ***comorbidities*** | Glaucoma | 122 (11.1%) | 44 (8.9%) | 0.18 | 10 (6.5%) | 156 (10.8%) | 0.09 |
| ***Cardiovascular comorbidities*** | Hypertension | 887 (80.6%) | 398 (80.1%) | 0.82 | 128 (82.6%) | 1,157 (80.2%) | 0.47 |
|  | Diabetes Mellitus | 267 (24.3%) | 196 (39.4%) | <0.01 | 75 (48.4%) | 388 (26.9%) | <0.01 |
|  | Dyslipidaemia | 704 (63.9%) | 319 (64.2%) | 0.93 | 117 (75.5%) | 906 (62.8%) | <0.01 |
|  | Dysrhythmias | 239 (21.7%) | 157 (31.6%) | <0.01 | 46 (29.7%) | 350 (24.3%) | 0.14 |
|  | Heart chronic failure | 126 (11.4%) | 68 (13.7%) | 0.21 | 23 (14.8%) | 171 (11.9%) | 0.28 |
|  | Ischaemic heart disease | 87 (7.9%) | 130 (26.2%) | <0.01 | 23 (14.8%) | 190 (13.2%) | 0.14 |
|  | Valvular heart disease | 66 (6.0%) | 34 (6.8%) | 0.52 | 20 (12.9%) | 80 (5.5%) | <0.01 |
| ***Musculoskeletal comorbidities*** | Arthrosis | 311 (28.2%) | 75 (15.1%) | <0.01 | 38 (24.5%) | 348 (24.1%) | 0.91 |
|  | Osteoporosis | 389 (35.3%) | 17 (3.4%) | <0.01 | 39 (25.2%) | 367 (25.4%) | 0.94 |
|  | Arthritis | 48 (4.4%) | 13 (2.6%) | 0.09 | 11 (7.1%) | 50 (3.5%) | 0.03 |
|  | Lupus | 2 (0.2%) | 1 (0.2%) | 0.93 | 1 (0.6%) | 2 (0.1%) | 0.17 |
|  | Vasculitis | 8 (0.7%) | 4 (0.8%) | 0.87 | 1 (0.6%) | 11 (0.8%) | 0.87 |
| ***Neurological comorbidities*** | Dementia | 117 (10.6%) | 32 (6.4%) | <0.01 | 3 (1.9%) | 146 (10.1%) | <0.01 |
|  | Stroke | 67 (8.0%) | 88 (13.5%) | <0.01 | 15 (9.7%) | 140 (9.7%) | 0.99 |
|  | Parkinson | 31 (2.8%) | 21 (4.2%) | 0.14 | 5 (3.2%) | 47 (3.3%) | 0.98 |
|  | Epilepsy | 35 (3.2%) | 13 (2.6%) | 0.54 | 12 (7.7%) | 36 (2.5%) | <0.01 |
|  | Multiple sclerosis | 1 (0.1%) | 0 (0.0%) | 0.50 | 0 (0%) | 1 (0.1%) | 0.74 |
| ***Psychiatric comorbidities*** | Alcohol abuse | 16 (1.5%) | 38 (7.6%) | <0.01 | 18 (11.6%) | 36 (2.5%) | <0.01 |
|  | Substance abuse | 4 (0.4%) | 2 (0.4%) | 0.91 | 3 (1.9%) | 3 (0.2%) | <0.01 |
|  | Anxiety | 233 (21.2%) | 58 (11.7%) | <0.01 | 37 (23.9%) | 254 (17.6%) | 0.06 |
|  | Depression | 260 (23.6%) | 51 (10.3%) | <0.01 | 35 (22.6%) | 276 (19.1%) | 0.30 |
|  | Bipolar disorder | 5 (0.5%) | 5 (1.0%) | 0.20 | 4 (2.6%) | 6 (0.4%) | <0.01 |
|  | Psychotic disorder | 11 (1.0%) | 4 (0.8%) | 0.71 | 3 (1.9%) | 12 (0.8%) | 0.18 |
| ***Respiratory comorbidities*** | COPD | 81 (7.4%) | 108 (21.7%) | <0.01 | 35 (22.6%) | 154 (10.7%) | <0.01 |
|  | Asthma | 83 (7.5%) | 9 (1.8%) | <0.01 | 12 (7.7%) | 80 (5.5%) | 0.26 |
| ***Endocrine comorbidities*** | Obesity | 270 (24.5%) | 113 (22.7%) | 0.44 | 55 (35.5%) | 328 (22.7%) | <0.01 |
|  | Thyroid disorder | 291 (26.4%) | 60 (12.1%) | <0.01 | 39 (25.2%) | 312 (21.6%) | 0.31 |
| ***Renal comorbidities*** | Renal chronic failure | 61 (5.5%) | 54 (10.9%) | <0.01 | 12 (7.7%) | 103 (7.1%) | 0.78 |
|  | Recurring urinary tract infection | 114 (10.4%) | 35 (7.0%) | 0.04 | 21 (13.5%) | 128 (8.9%) | 0.06 |
| ***Neoplastic comorbidities*** | Any active neoplasia | 95 (8.6%) | 98 (19.7%) | <0.01 | 42 (27.1%) | 151 (10.5%) | <0.01 |
|  | Breast | 28 (2.5%) | 1 (0.2%) | <0.01 | 7 (4.5%) | 22 (1.5%) | <0.01 |
|  | Prostate | 0 (0%) | 35 (7.0%) | <0.01 | 8 (5.2%) | 27 (1.9%) | <0.01 |
|  | Skin | 16 (1.5%) | 8 (1.6%) | 0.81 | 2 (1.3%) | 22 (1.5%) | 0.82 |
|  | Colorectal | 13 (1.2%) | 9 (1.8%) | 0.32 | 7 (4.5%) | 15 (1.0%) | <0.01 |
|  | Bladder | 2 (0.2%) | 18 (3.6%) | <0.01 | 6 (3.9%) | 14 (1.0%) | <0.01 |
|  | Lung | 6 (0.5%) | 9 (1.8%) | 0.02 | 5 (3.2%) | 10 (0,7%) | <0.01 |
|  | Cervix | 1 (0.1%) | 0 (0%) | 0.50 | 0 (0%) | 1 (0.1%) | 0.74 |
|  | Liver-Gastrointestinal-Pancreas | 6 (0.5%) | 4 (0.8%) | 0.54 | 0 (0%) | 10 (0.7%) | 0.3 |
|  | Renal | 3 (0.3%) | 6 (1.2%) | 0.02 | 3 (1.9%) | 6 (0.4%) | 0.02 |
|  | Endometrial | 2 (0.2%) | 0 (0%) | 0.34 | 1 (0.6%) | 1 (0.1%) | 0.05 |
|  | Leukaemia | 5 (0.5%) | 7 (1.4%) | 0.04 | 2 (1.3%) | 10 (0.7%) | 0.41 |
|  | Lymphoma | 11 (1.0%) | 6 (1.2%) | 0.71 | 1 (0.6%) | 16 (1.1%) | 0.59 |
|  | Other neoplasia | 4 (0.4%) | 4 (0.8%) | 0.25 | 2 (1.3%) | 6 (0.4%) | 0.14 |
| Abbreviations: HIV, Human Immunodeficiency Virus; COPD, Chronic Obstructive Pulmonary Disease. | | | | | | | |
